# Analysis of plant LTR-retrotransposons at the fine-scale family level reveals individual molecular patterns

**DOI:** 10.1186/1471-2164-13-137

**Published:** 2012-04-16

**Authors:** Douglas S Domingues, Guilherme MQ Cruz, Cushla J Metcalfe, Fabio TS Nogueira, Renato Vicentini, Cristiane de S Alves, Marie-Anne Van Sluys

**Affiliations:** 1 GaTE Lab, Depto. de Botânica, Inst. de Biociências, Universidade de São Paulo, Rua do Matão, 277, 05508-090 São Paulo, Brazil; 2 Depto. de Genética, Inst. de Biociências, Universidade Estadual Paulista, campus de Botucatu, Distrito de Rubião Jr., s/n, 18618-000 Botucatu, Brazil; 3 Systems Biology Laboratory, Centro de Biologia Molecular e Engenharia Genética, Universidade Estadual de Campinas, Av. Cândido Rondon, 400, 13083-875 Campinas, Brazil; 4 Plant Biotechnology Laboratory, Instituto Agronômico do Paraná, Rod. Celso Garcia Cid (PR-445), km375, 86047-902 Londrina, Brazil

**Keywords:** LTR Retrotransposons, Sugarcane, Genome, FISH, Epigenetic, Small RNA

## Abstract

**Background:**

Sugarcane is an important crop worldwide for sugar production and increasingly, as a renewable energy source. Modern cultivars have polyploid, large complex genomes, with highly unequal contributions from ancestral genomes. Long Terminal Repeat retrotransposons (LTR-RTs) are the single largest components of most plant genomes and can substantially impact the genome in many ways. It is therefore crucial to understand their contribution to the genome and transcriptome, however a detailed study of LTR-RTs in sugarcane has not been previously carried out.

**Results:**

Sixty complete LTR-RT elements were classified into 35 families within four *Copia *and three *Gypsy *lineages. Structurally, within lineages elements were similar, between lineages there were large size differences. FISH analysis resulted in the expected pattern of *Gyps*y/heterochromatin, *Copia*/euchromatin, but in two lineages there was localized clustering on some chromosomes. Analysis of related ESTs and RT-PCR showed transcriptional variation between tissues and families. Four distinct patterns were observed in sRNA mapping, the most unusual of which was that of *Ale1*, with very large numbers of 24nt sRNAs in the coding region. The results presented support the conclusion that distinct small RNA-regulated pathways in sugarcane target the lineages of LTR-RT elements.

**Conclusions:**

Individual LTR-RT sugarcane families have distinct structures, and transcriptional and regulatory signatures. Our results indicate that in sugarcane individual LTR-RT families have distinct behaviors and can potentially impact the genome in diverse ways. For instance, these transposable elements may affect nearby genes by generating a diverse set of small RNA's that trigger gene silencing mechanisms. There is also some evidence that ancestral genomes contribute significantly different element numbers from particular LTR-RT lineages to the modern sugarcane cultivar genome.

## Background

Plant genome evolution is closely associated with polyploidy and gene amplification, the most highly amplified genes being Long Terminal Repeat retrotransposons (LTR-RTs) [1,2]. LTR-RT proliferation is regulated by the genome at both the transcriptional and post-transcriptional level [3]. LTR-RTs, like other Transposable Elements (TEs), can not only affect a genome by expansion by proliferation, but also by, for example, providing a template for recombination, inserting into coding regions and disrupting gene expression, or affecting the transcription of neighboring genes (reviewed by [4]).

Based on the massive amounts of genomic sequence data released in the last 15 years, phylogenetic analysis of plant LTR-RTs has identified distinct evolutionary lineages within *Gypsy *and *Copia*, the two main plant LTR-RT superfamilies [5]. These lineages are widespread in both monocot and eudicot genomes. Uncovering of this fine structuring within *Gypsy *and *Copia *plant LTR-RTs has lead to a better understanding of the diversification of LTR-RTs and shed light on their structure and genomic distribution [6-8].

Sugarcane is an important crop worldwide, being a major source of sugar, and is also increasingly being used for the production of renewable energy sources such as ethanol. Despite its economic importance, the sequencing of the sugarcane genome is at the pilot stage [9]. Modern cultivars are highly polyploid and have one of the largest (10Gb) and most complex crop genomes, and chromosome numbers varying between 100-130 [10].

There are a few reports on transposable elements in sugarcane sequences [11-14] but there is no detailed study on sugarcane LTR-RTs, their phylogenetic classification, and potential impact on genomic distribution and transcriptional activity. In addition, the importance of accurate and complete TE annotation is increasingly recognized as a priority in plant genome sequencing projects to minimize the inaccuracy of gene annotation and facilitate functional gene studies [15].

To set the basis for future genome interpretation, LTR-RTs in the sugarcane genome were characterized using available public resources and BAC sequences from the BIOEN project [16]. We examined the structure, genomic distribution, phylogenetic diversity, transcriptional activity and regulation of sugarcane LTR-RTs. This study reveals that within well-defined phylogenetic lineages, that while TEs within LTR-RTs families are structurally similar, they have distinct transcriptional and regulatory signatures. Taken together, these results support the growing evidence that LTR-RTs contribute to genomic diversity, but with a wide range of potential outcomes.

## Results

### Characterization of LTR retrotransposons in sugarcane: phylogeny and structural features

**Figure 1:**
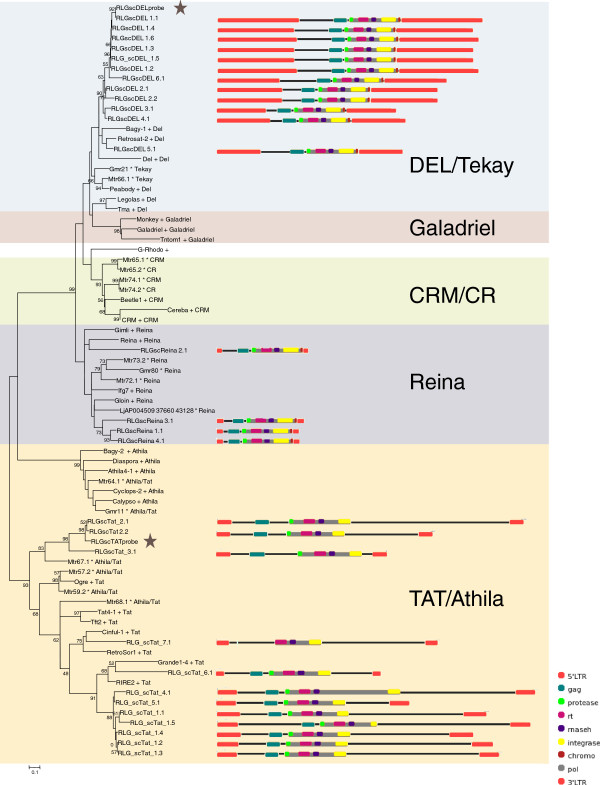
**Neighbour-joining (NJ) phylogenies of *Copia *and *Gypsy *families**. NJ phylogeny of *Gypsy *families based on reverse transcriptase, with schematic representations of sugarcane elements. Sequences from the Gypsy database [8] are denoted with a plus symbol, those from Du et al. [7] with a star. Robustness of the nodes was estimated by 500 bootstrap replications. Bootstrap values below 50% are not shown. Lineages are indicated by names and colored blocks. A star indicates the sequences used as probes for fluorescent *in situ *hybridization (Figure 3). Schematic representations were created using domain draw [17]. A scale and a key for the domains represented in the schematic representations are shown in the bottom right hand corner. Abbreviations and color coding of domains: LTR = long terminal repeat (orange); gag = Gag (dark green); protease = Protease (light green); rt = Reverse Transcriptase (pink); rnaseh = Ribonuclease H (purple); integrase = Integrase (yellow); chromo = Chromodomain (brown); env = Envelope (brown); pol = Polyprotein (grey).

**Figure 2:**
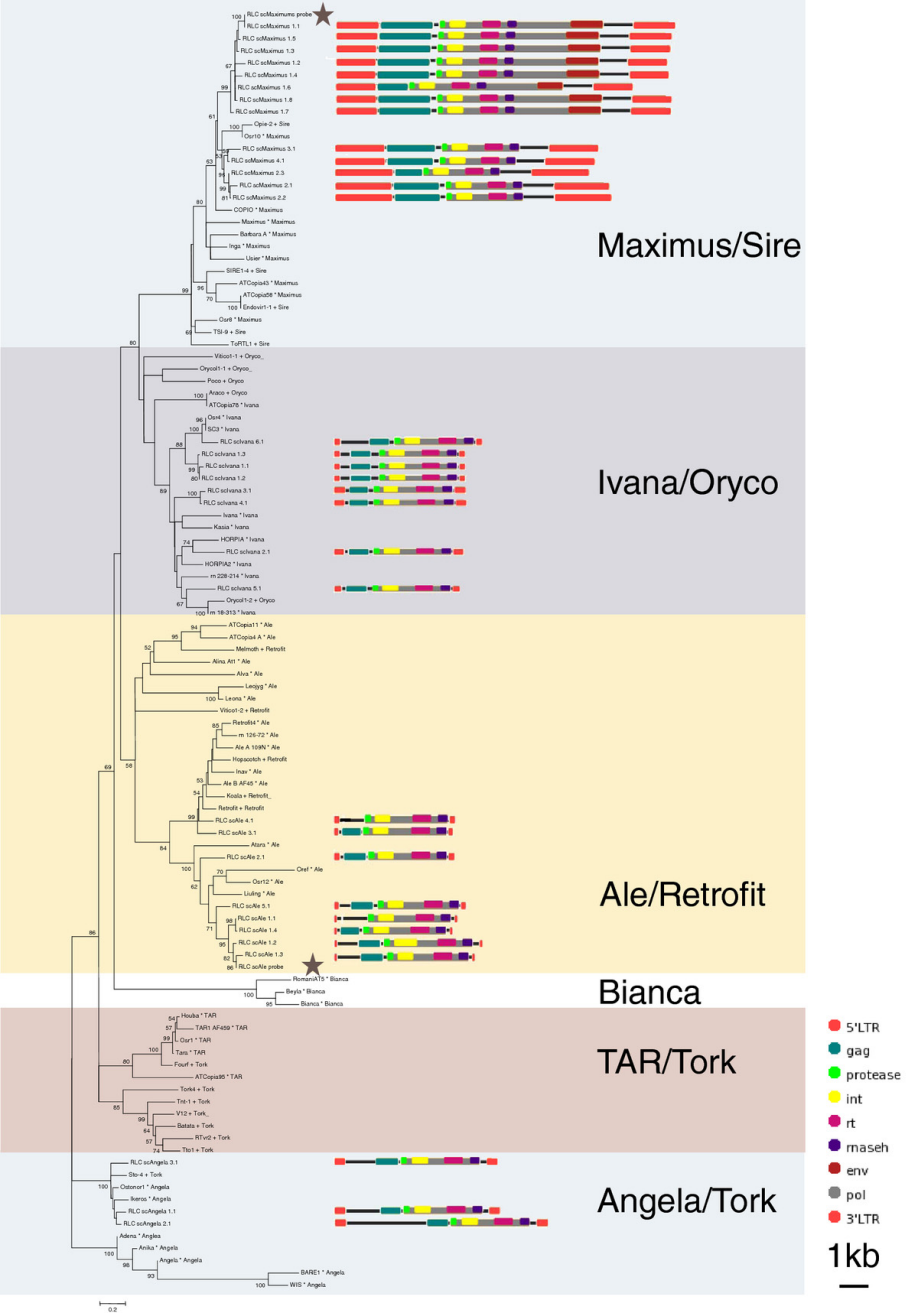
**Neighbour-joining (NJ) phylogenies of *Copia *and *Gypsy *families**. NJ phylogeny of *Copia *families based on reverse transcriptase, with schematic representations of sugarcane sequences. Sequences from the Gypsy database [8] are denoted with a plus symbol, those from Wicker and Keller [6] with a star. Robustness of the nodes was estimated by 500 bootstrap replications. Bootstrap values below 50% are not shown. Lineages are indicated by names and colored blocks. A star indicates the sequences used as probes for fluorescent *in situ *hybridization (Figure 3). Schematic representations were created using domain draw [17]. A scale and a key for the domains represented in the schematic representations are shown in the bottom right hand corner. Abbreviations and color coding of domains: LTR = long terminal repeat (orange); gag = Gag (dark green); protease = Protease (light green); rt = Reverse Transcriptase (pink); rnaseh = Ribonuclease H (purple); integrase = Integrase (yellow); chromo = Chromodomain (brown); env = Envelope (brown); pol = Polyprotein (grey).

Plant LTR-RT evolutionary lineage names are not used consistently within the literature [6-8], we therefore chose to include sequences from more than one source, so that we could directly compare our results with published data. Our inferred evolutionary histories suggest that, at least for the sequences we analyzed within *Gypsy *superfamily, the *DEL *lineage is equivalent to *Tekay*, and within the *Copia *superfamily, the *Maximus *lineage is equivalent to *Sire*, *Ale *to *Retrofit *and *Ivana *to *Oryco *(Figures 1 and 2; Additional file 1). The relationship between the *TAR*, *Angela*, *Tork *and *Bianca *lineages is more problematic. While the *Bianca *lineage is not included in the GyDb [8]*Tork *appears to be the *Angela *and *TAR *lineages combined (Figure 2; Additional file 1: Figure S2).

Of the sixty full-length sequences extracted from sugarcane BAC sequences, thirty-two sequences were assigned to the *Copia *superfamily and twenty-eight to the *Gypsy *superfamily (Table 1; Figures 1 and 2). We identified four of the six major plant evolutionary *Copia *lineages described by Wicker and Keller [6] in the sugarcane genome (Figure 2; Additional file 1: Figure S2), and three of the six *Gypsy *lineages described by Du et al. [7] and the GyDB [8] (Figure 1; Additional file 1: Figure S1). In terms of sequence numbers, the *Maximus *lineage was the most highly represented within the *Copia *superfamily, and the *Del *and *Tat *lineages were equally represented within the *Gypsy *superfamily. Based on sequence identity within the LTRs, the elements were classified into 35 families (Table 1) [5]. In order to simplify reading of the text we have referred to these families as simply '*Ale1*' for example, rather than the by the full name '*RLC_scAle1'*.

**Table 1:** General features of sugarcane LTR-RT lineages

Superfamily/Lineage	Size (kb)	LTR (bp)	Families	Sequences
*Copia*				

Ale	4.7-5.9	116-238	5	8

Angela	6.5-8.5	434-461	3	3

Ivana	5-5.9	238-454	6	8

Maximus	10.2-13.6	1607-2004	4	13

*Total Copia*			18	32

*Gypsy*				

DEL	11.3-16.7	2762-5139	6	12

Reina	5.1-5.7	315-416	4	4

TAT	9.2-17.7	458-1345	7	12

*Total Gypsy*			17	28

Overall Total			35	60

We were able to identify all the internal coding domains (gag, aspartic protease, reverse transcriptase, integrase and RNAseH) in all the sugarcane sequences, apart from the gag domain in *RLG_scTat_7.1 *(Figure 1). In addition, a putative envelope domain was identified in elements from the *Maximus1 *family (Figure 2) and a chromodomain in all *DEL *and *Reina *elements (Figure 1) [8].

There was a general pattern in overall size of elements from lineages in each of the two superfamilies. In both superfamilies, there was one lineage that is very large (*Maximus *and *Del*, 10.2-16.7 kb), and at least one lineage that is comparatively smaller (*Ivana*, *Ale *and *Reina*, 4.7-5.9 kb) (Figures 1 and 2). Within the *Copia *superfamily, there is a lineage that is an in-between size (*Angela *at 6.5-8.5 kb), while within the *Gypsy *superfamily, the sequences of the *Tat *lineage (9.2-17.7 kb) are more comparable in size to those of the *DEL *lineage (11.3-16.7 kb) (Figures 1 and 2). Differences in total length were chiefly due to differences in LTR size, and the presence and size of spacer regions between the internal coding domain and the LTRs, rather than insertions within the gag/pol coding regions (Figures 1 and 2). The exception is the *Tat *elements, which contain insertions within the pol region, but in most cases between actual coding domains (Figure 1).

### Distribution of LTR retrotransposons in metaphases

**Figure 3:**
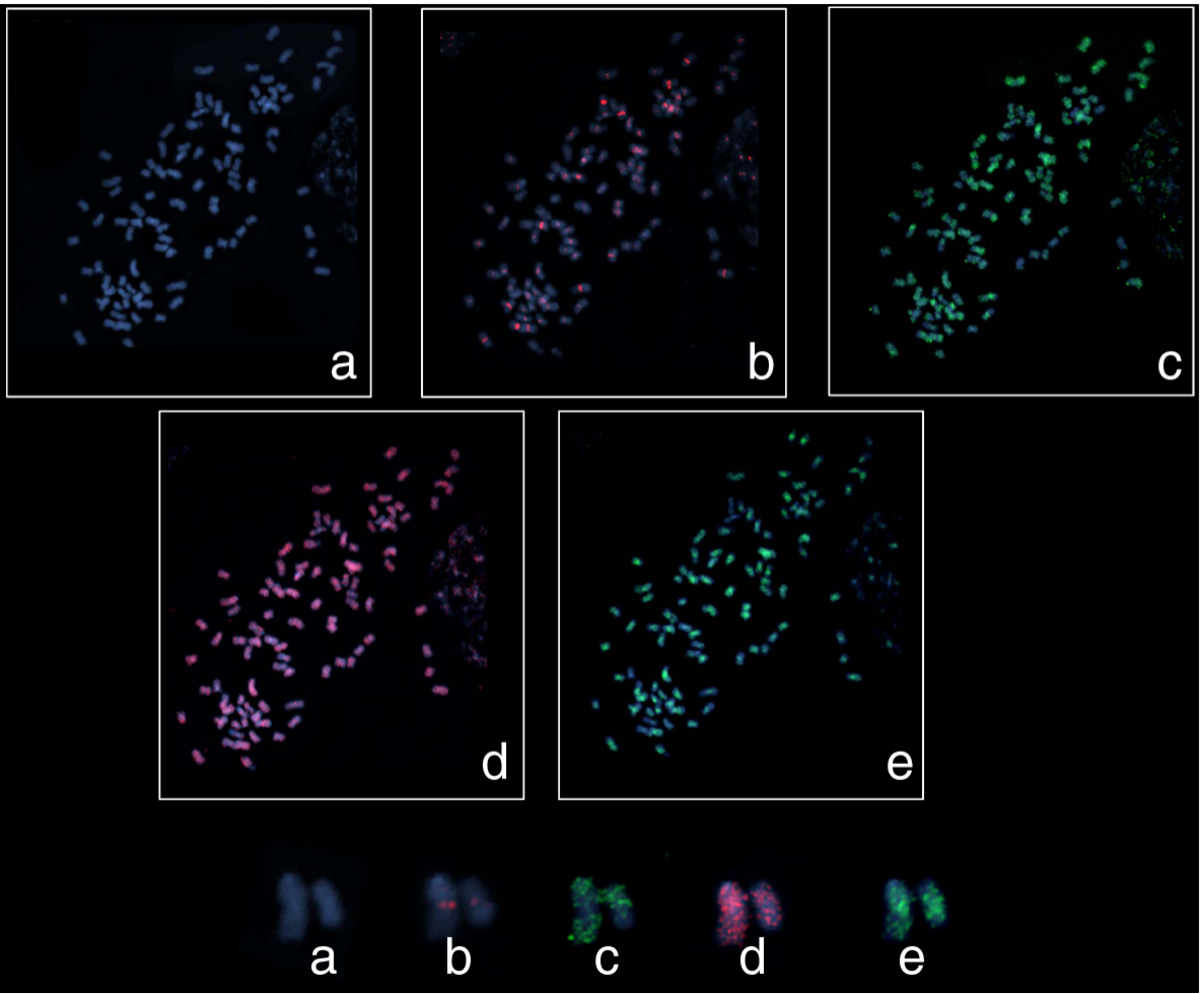
**Fluorescent *in situ *hybridization patterns observed for sugarcane LTR-RTs**. A pair of representative chromosomes is shown underneath the metaphase spreads. Chromosomes are stained with DAPI, probes were labelled with Digoxigenin (DIG) and detected with Anti-DIG-Rhodomine (red signal) or with Biotin and detected with NeutrAvidin-Oregon Green (green signal) **(a) **DAPI counterstaining only. **(b) **BAC SCHRBa_239_N21 which contains known sugarcane centromeric repeats **(c) **scAle probe (euchromatic pattern). **(d) **scMaximus probe (dispersed pattern) **(e) **scDELprobe (centromeric/pericentromeric pattern). The LTR-RT probes were 1.9-2.9 kb and included the reverse transcriptase domain.

Given that transposable elements can be distributed throughout a genome, it is difficult to distinguish between random and real signals from a probe derived from a transposable element. The probe for the *Del *lineage was therefore prepared twice, one probe labeled with DIG and the other with biotin, and hybridized separately to the same slide before and after stripping. The distribution of the *Del *probe was compared on the same 10 metaphases visualized with anti-digoxigenin-rhodamine (Red) or with NeutrAvidin-Oregon Green-488 (Green) (Additional file 2). The distribution of the probe signal was similar in all 10 metaphases, and therefore the signal was considered to be valid. Similar *in situ *hybridization conditions were used for all subsequent experiments. A BAC clone (SCHRBa_239_N21), identified by our group [16], contains known sugarcane centromeric repeats [18,19]. This BAC clone hybridized to the middle region of the chromosome, which consists mainly of centromeric specific sequences (Figure 3b).

Seven LTR-RT probes were used, three of which are from *Gypsy *superfamily members and four from *Copia *superfamily members, representing all the major lineages already described. No convincing signal was obtained for the *Reina1, Ivana1 *and *Angela1 *probes, using the same conditions that were used for the other LTR-RT probes.

The probe from one of the two *Gypsy *lineages, *Del1*, localized almost exclusively in a broad band around the centromeres. This observation suggests that it is a heterochromatic or pericentromeric specific element (Figure 3e). The second *Gypsy *element examined, *Tat2*, displays a generally strong broad dispersed pattern, but with some concentration along some chromosome arms (data not shown).

Two *Copia *probes were found dispersed along the chromosome arms and none localized exclusively at or near the centre of the chromosomes. The *Ale1 *lineage probe was found in high concentrations along particular chromosome arms (Figure 3c). Signals from the *Maximus *lineage probe were generally dispersed (Figure 3d).

### Transcriptional activity of sugarcane LTR retrotransposons

**Figure 4:**
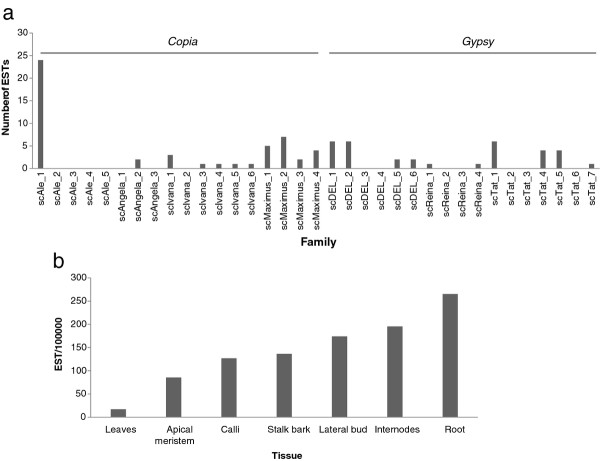
**Transcriptional activity of sugarcane LTR-RTs**. ESTs from the cultivar SP80-3280 available from NCBI were assigned to an LTR-RT family, using the criteria described by Wicker et al. (2007) [5]. **(a) **Total normalized number of ESTs assigned to each LTR-RT family and **(b) **Total normalized number of ESTs by tissue type.

We associated 84 ESTs from the sugarcane cultivar SP80-3280 related to full-length LTR retrotransposons. The largest number of transcripts was identified from the root libraries (Figure 4b), followed by the internode, lateral bud and calli libraries.

The *Copia *superfamily was more highly represented in the EST database than the *Gypsy *superfamily, with 51 and 33 sequences, respectively (Figure 4a). Previously described full length cDNA sequences [20] were assigned to 8 of the 35 families we identified from BAC sequences (Additional file 3). In this study, no ESTs were identified for 14 families (Figure 4a). We assigned ESTs to another 14 families not previously described as transcriptionally active [11,20] (Figure 4a), however for one family, *Ale3*, for which a previously described cDNA sequence was assigned, no ESTs were identified.

RT-PCR analysis confirmed the transcriptional activity of sugarcane LTR-RTs in leaves and lateral buds. We also experimentally confirmed transcriptional activity for five new families: *Ale2*, *Ivana6*, *Del2, Reina1 *and *Reina3*.

**Figure 5:**
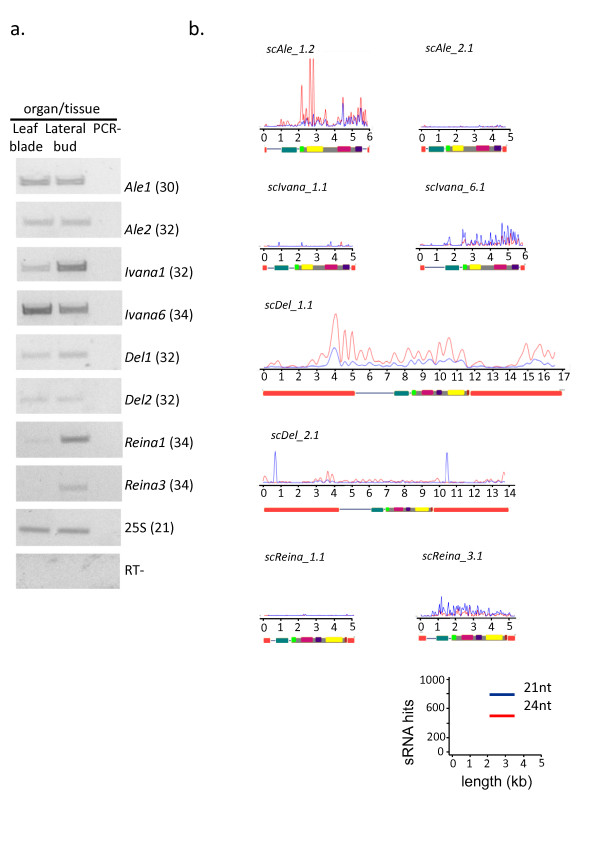
**Mapping of small RNAs in confirmed transcriptionally active LTR-RTs families**. **(a) **Sugarcane TEs are transcriptionally active in leaf and bud tissues. Expression profiles of selected TEs in leaf blade and developing lateral buds of the sugarcane hybrid SP80-3280. Ribosomal *25S *gene (Sc25S) was used as a loading control. RT- (reaction without RT) and PCR- (reaction without cDNA) are shown as negative controls. Numbers in parentheses represent PCR cycles for each amplicon. **(b)**. Mapping of sRNA within 8 LTR-RT families. 20-22nt sRNAs are represented as blue lines, 23-25nt sRNAs as red lines. A scaled schematic (also shown in Figures 1 and 2), is shown below each sRNA map.

The most represented family in the EST data, *Ale1*, had an intense band, confirming its transcriptional activity, and had the same intensity of signal in both leaves and lateral buds. Transcriptional patterns differed between tissues and LTR-RT families (Figure 5a). The *Ivana *and *Reina *lineages illustrate that individual families have distinct transcriptional patterns: for instance, the *Ivana6 *signal is more intense than that of *Ivana1*; moreover, *Ivana1 *has higher transcriptional activity in lateral buds, while *Ivana6 *is more active in leaves. *Reina1 *and *Reina3 *are both transcriptionally active in lateral buds, but clear differences in band intensity can be observed (Figure 5a).

### Distribution of small RNAs among LTR retrotransposon lineages

We divided the sRNA population into two major classes: 20-22nt and 23-25nt sRNA sequences. We refer hereafter to these classes as 21-nt class (20-22nt) and 24-nt class (23-25nt). In this analysis we allowed a 0-2 nt mismatch between the sRNA and LTR-RT reference sequences, in which 25% and 22% of the 21 and 24 ntRNAs class, respectively, showed a perfect match with the LTR-RTs sequences. All identified lineages had at least one family with sRNA from both classes mapped to LTR-RTs. In the Copia superfamily, all families within the Maximus lineage had more than 8000 sRNAs mapped, mostly belonging to the 24-nt class (Additional file 4: Figure S5 and Additional file 5). These elements had the highest sRNA counts, about 1.5 to 5.5 fold higher than other lineages and these counts mapped chiefly to the LTRs (Additional file 4: Figure S5). Del1 is the only representative of the Gypsy superfamily that has comparable sRNA counts. The Ivana and Reina lineages were the lineages with the least number of sRNA counts (Additional file 5). Within the Ivana lineage, the element RLC_scIvana6.1 had the highest number of sRNA matches, most of which belong to the 21-nt class (Additional file 5). The same pattern was observed in RLG_Reina3.1. The remaining Ivana and Reina families had lower sRNA counts.

To evaluate whether sRNAs preferentially matched specific regions of each LTR-RT, we compared the sRNA distribution within LTRs and coding domains (Additional file 4: Figure S1-S7). Various distribution patterns, both within the LTRs and the coding domains were observed. Families of the Maximus lineage and Del1 had the high 24-nt sRNA counts within the LTRs. The 5' region of both LTRs from Del2 and Del3 families had a peak of 21-nt sRNAs. Most LTR-RT families did not have high sRNA counts within the coding domains, but some cases of high counts of sRNAs were noted. The Ale1 family had > 1000 counts of the 24-nt class sRNAs within the integrase and protease domains and the Tat2 family had > 6000 counts of the 24-nt class sRNAs within the RnaseH domain (Additional file 4: Figure S4, Figure S3). All other cases of high counts in coding domains are related to 21-nt class sRNAs, as seen in the Ivana6 and Reina3 families, both with around 300 counts. Interestingly, Angela2 had peaks of > 200 24-nt sRNA counts in a 3.5 kb non-coding region between the 5'LTR and the gag domain (Additional file 4: Figure S5).

## Discussion

We characterized LTR-RTs in the sugarcane genome using BAC sequences available from the BIOEN project and publicly available genomic and EST resources, which allowed us to provide the most complete overview of the structural and phylogenetic diversity of these elements to date. The 35 LTR-RT families identified belong to four lineages from the *Copia *superfamily and to three from the *Gypsy *superfamily. Among all these families we observed distinct patterns of differences in structural features, chromosomal distribution, transcriptional activity, and sRNAs targeting.

### Sugarcane LTR-RTs are structurally diverse and belong to evolutionary lineages shared by monocots and eudicots

Previous surveys of LTR-RTs in plants defined six major common evolutionary *Copia *lineages [6] and six major common evolutionary *Gypsy *lineages [8]. *Bianca *was the only *Copia *superfamily lineage in which sugarcane LTR-RTs were not found. Absence of *Bianca *in the sugarcane genome dataset analyzed may be explained by its relatively low representation in other plants. Indeed, rice has few *Bianca *families [6] and soybean has none [7].

We also did not find sugarcane LTR-RTs elements from the *Galadriel *and *CRM *lineages in *Gypsy *superfamily. The *Galadriel *lineage seems to be an under-represented lineage among monocots, since there are only reports of *Galadriel *elements in banana [21]. *CRM *elements are also minor components of *Gypsy *retrotransposons in the model grass rice [7], which may explain its low representation in sugarcane.

Although very little sequence of the sugarcane genome is available, the number of LTR-RT families we identified was comparable to previous large scale LTR-RTs analyses in BAC clones from *Medicago truncatula *(232 Mb) [22] and *Capsicum annuum *(35.6 Mb) [23]. This finding suggests that we have a good overview of the diversity of sugarcane LTR-RTs.

### Sugarcane LTR-RT families have distinct chromosomal distributions

Transposable elements are not distributed randomly along eukaryotic chromosomes [24]. In particular, *Gypsy *elements tend to be found in heterochromatic regions, *Copia *elements are more dispersed throughout the genome [25,26]. In addition, heterochromatic and pericentromeric regions are enriched in TEs, such as those of the *CR/CRM *and *Tat *lineages, compared with euchromatic regions [27-29]. Using fluorescence in situ hybridization (FISH), we examined the distribution of representatives from seven lineages identified within the *Gypsy *and *Copia *superfamilies in sugarcane chromosomes. No detectable FISH signal was obtained for the *Angela*, *Ivana *and *Reina *lineages; in the case of *Ivana *preliminary estimates from the R570 BAC library suggests that there is approximately 50 copies of *Ivana1 *per haploid genome in sugarcane (data not shown), which suggests that the lack of signal is due to low copy number. We suspect that this observation may be also valid for *Angela *and *Reina*.

The two *Copia *probes, *Ale1 *and *Maximus1*, had the expected euchromatic patterns, but they were distinctly different. The signals from the *Ale *probe had localized clusters along particular chromosome arms, while those of the *Maximus1 *probe were widespread and dispersed, without any signs of concentration in particularly regions (Figure 3d). It has been suggested that as TEs accumulate in a genome, even if they insert randomly, they are more likely to insert within other TEs, forming clusters of TEs. Because these clusters of TEs are most likely selectively neutral, they will be free to expand [24]. The *Ale *distribution pattern observed in sugarcane is reminiscent of such clusters of TEs.

The sugarcane BAC SCHRBa 239_N21 used as a centromeric-specific probe in this study contains the SCEN repeat interspersed with LTR-RTs from the *Tat *lineage [16]. Previous studies in sugarcane [18,19] identified a centromere-specific repeat (SCEN) and centromere associated LTR-RTs, classified as *CR/CRM*-like. We did not identify any full length LTR-RTs from the *CR/CRM *lineage in this study, but sequence from a related *Gypsy *lineage, *Del1*, did hybridize to a broad region around and within the centromeric region (Figure 3b and 3e). *Del *lineage sequences have been identified at other plant centromeres [28], however our results suggest that the *Del *sequence we used as a probe is not strictly centromeric specific, but is rather preferentially found in and around heterochromatic regions of the centromeric repeats. The *Tat *sequences identified in the BAC SCHRBa 239_N21 clustered with the *Tat1*, *Tat4*, *Tat5 *and *Tat6 *families, (data not shown) which is quite distinct from the *Tat2 *family (Figure 1). The *Tat *FISH probe used is most closely related to the *Tat2 *sequences (Figure 1) and had a clusters of localization along some chromosomes, like those seen with the *Ale1 *probe, but with much more intense signals.

Modern sugarcane cultivars are interspecific hybrids between *Saccharum officinarum *and *S. spontaneum *and backcrossed with *S. officinarum *[30]. The resulting genomes are highly polyploid, 70-80% of which is from *S. officinarum*, 10-23% from *S. spontaneum*, the rest being recombinants [10,31]. The derivation of the modern sugarcane genome suggests that, rather than being the result of TEs inserting into clusters of TEs, the distribution of *Tat2 *and *Ale1 *may be the result of differential transmission of TEs from the parental genomes. Future work using genomic in situ hybridization [31] combined with FISH should enable us to distinguish between these two hypotheses.

### Transcriptional activity of LTR-RTs and their associated sRNAs

Transposable elements make up a substantial proportion of plant genomes, and are often transcriptionally active. A very stringent search for sugarcane ESTs associated with TEs confirmed that transcriptional activity appears to be a general feature of some sugarcane LTR-RTs, as reported for other monocots [32,33]. Since we do not have detailed information about cDNA library preparation of EST data, we cannot identify if transcribed sequences were in sense or antisense orientation. Interestingly, within each lineage, the number of transcripts mapped to each family was variable (Figure 4a). The most striking example is the *Ale *lineage, which has the highest number of transcripts, all of which mapped to a single family, *Ale1*. There is a similar, but not as definitive pattern in all other lineages, except for *Maximus*, where all families have similar numbers of transcripts.

In terms of tissue specificity (Figure 4b), the highest number of LTR-RT associated transcripts was identified from the root libraries, followed by the internode, lateral bud and calli libraries. A previous macroarray analysis identified calli as the tissue with the highest number of TEs being expressed in sugarcane [20], however in this study only calli, apical meristem, leaf roll and flower were analyzed. These differences in expression according to tissue, in particular that leaf is the tissue with least number of ESTs identified, may help guide future studies of transposable elements in sugarcane.

As they are potentially highly mutagenic, the activity of LTR-RTs is usually controlled by the host genome through the siRNA machinery. The specificity of this response is achieved by a surveillance system that detects aberrant RNA. The proliferative nature of TEs makes them prone to insert in the genome in such way that both sense and anti-sense transcripts are produced, generating dsRNA, and activating the siRNA system.

Two main classes of siRNAs are generated, the 21-nt class regulates post-transcriptionally related mRNAs while the 24-nt class is involved in RNA-dependent DNA methylation (RdDM) and heterochromatin maintenance and therefore suppresses gene expression at transcriptional level [34].

Previous studies mapping sRNAs to LTR-RTs in wheat and maize genomes [35,36], presented an overall study of TE superfamilies and described a pattern of concentration of 24nt sRNA in the LTRs. Our work, which focused on mapping to individual LTR-RT elements, identified distinct patterns of sRNA targeting within sugarcane LTR-RT lineages and families.

The previously described '24nt LTR' pattern was observed for all references sequences in the *Maximus *families, and for *Del1 *and *Tat3 *(Additional file 5). For all other reference sequences a different type of pattern was observed. For almost half of the families (18 out of 33) very few sRNAs(< 2000 counts) were mapped to the reference copy (Additional file 5). Two other patterns were observed, one in which high numbers of 21nt sRNAs mapped along the coding region, represented by *Ivana6 *and *Reina3 *(Additional file 5), and one in which a very large number of 24nt sRNAs mapped within the coding region, seen only in *Ale1*.

Very few mapped sRNAs indicates that elements from those families either are not transcriptionally active, or they are very recently activate and have not yet triggered the host small RNA-dependent silencing machinery. If they are not transcriptionally active, silencing may be being maintained by ancient methylation. A previous study has shown that ~63% of methylated regions were maintained without persistent targeting by sRNAs [37], explaining the absence of both 21 and 24nt sRNAs.

The high numbers of 21nt sRNAs mapped to the coding region of some families suggests that they are being regulated primarily post-transcriptionally. This has been previously reported for MITEs [36] and may indicate recent activation of transcription, pior to triggering of the RdDM machinery.

The unique pattern observed in the *Ale1 *family suggests that the RdDM machinery is guiding methylation to the coding region of the element, not the promoter region. The "body-methylated gene" concept was first described in plants in 2006, when Zhang and collaborators showed that over one third of *Arabidopsis *expressed genes were methylated in the coding region, but not in the promoter region [37]. The *Ale1 *family has a distinct profile, it has a 'body-gene'-like sRNA pattern, it is the most transcriptionally active LTR-RT in sugarcane and is concentrated in euchromatic regions and gene-rich BAC sequences ([12] and Figure 3c). At this point it is difficult to make conclusions from these observations, however these results are intriguing and warrant further investigation.

## Conclusions

This is the first study to perform a concomitant survey of phylogenetic diversity, chromosomal distribution, structure, transcriptional activity and interaction with sRNAs of LTR-RTs in a plant genome. We assigned 60 LTR-RT elements to 35 families within four *Copia *and three *Gypsy *lineages. Two lineages, one *Copia *and one *Gypsy *lineage, showed distinct patterns of signal clustering along some chromosomes in the FISH analysis. Given that the modern sugarcane cultivars are hybrids with highly unequal contributions from the ancestral genomes, the FISH patterns suggest that for these lineages there has been higher numbers of elements from one ancestral genome than the other. For the transcriptional and sRNA mapping analyses we chose to analyze at the family level. Individual families had distinct transcript and sRNA mapping profiles, suggesting that they are differentially expressed and regulated. The *Ale1 *family was particularly unusual in that it had 'body-gene'-like sRNA pattern, it is the most transcriptonally active LTR-RT in sugarcane and is concentrated in euchromatic regions. Overall, our results indicate that LTR-RTs could impact the genome in different ways at the family levels.

## Methods

### Identification and retrieval of sugarcane LTR retrotransposon sequences

All BACs used are from the R570 sugarcane cultivar library [38]. BACs sequenced for the BIOEN Project [16] and public sugarcane BAC sequences available at the National Center for Biotechnology Information (NCBI) website as at 01/02/2011 were screened for full-length LTR elements using LTR_STRUC [39] with the most thorough stringency (1). Sixty sequences were retrieved and provisionally assigned to the *Gypsy *or *Copia *superfamily by submission against cores in the Gypsy Database (GyDB) [8] using BLASTX. To determine whether the sequences were complete elements, we identified target site duplications (TSDs) by submitting the full length sequences as a query and subject to a blast2seq [40] on the NCBI website.

### Phylogenetic analysis

Sugarcane LTR-RTs, including the probes used for fluorescence *in situ *hybridization, were assigned to previously described plant LTR lineages [6-8] by phylogenetic analysis using the translated reverse transcriptase (RT) domain excised from all the sugarcane LTR-RTs and published RT sequences.

For both phylogenies we downloaded RT alignments from the Gypsy database (GyDB) [8], and removed non-plant sequences. *Gypsy *sequences were also taken from Du et al. (2010) [7], *Copia *sequences were taken from Wicker and Keller (2007) [6]. All sequences were renamed to reflect published lineage names. Sequences were aligned using MUSCLE with default settings [41] and manually adjusted by eye. The optimal model of amino acid substitution was estimated using MEGA5 [42] with default settings. Neighbor-joining and maximum-likelihood phylogenies were estimated with MEGA5 [42] using the highest-ranked substitution model available and a bootstrap of 500 replicates.

### Assignment to Families within Lineages and naming of sequences

Sugarcane LTR-RTs were assigned to families within lineages on the basis of 80% sequence identity in at least 80% of their LTRs [5]. Although previous reports assign names to some sugarcane LTR-RT families [11,14,20], we opted to standardize the name of sugarcane LTR-RT sequences, using a more straightforward strategy, based in the proposed universal classification of TEs by Wicker et al. (2007) [5]. Sequences were named 'RLC' (*Copia*) or 'RLG' (*Gypsy*), 'sc' for 'sugarcane', the lineage name e.g. '*Ale*', the family number e.g. '1', then each sequence within a family was numbered sequentially. For example '*RLC_scAle_1.1*' is the first sequence named within the *Ale *lineage, family 1, superfamily *Copia*.

### Analysis of the structure of Sugarcane LTR-RTs

Coding domains were identified using Pfam, or by alignment with MUSCLE [41] against the domain alignment from the GyDb [8]. Full-length sequences were aligned and analyzed using BioEdit [43], using the toggle translate option so that we could align the coding domains as well as the LTRs, TSDs, and the regions between the LTRs and the coding domains. LTRs were identified by submitting the sequence of the entire sugarcane LTR-RT as both a query and subject to a MEGABLAST [40] analysis. The beginning of the LTRs, regions between the LTRs and the coding domains, and the TSDs were manually aligned in BioEdit [43]. Co-ordinates of the beginning of all features of each element were recorded in an Excel table and the information submitted to domain draw [17] to create a schematic representation of each sugarcane LTR-RT.

General features of each sequence, as element size, LTR size, Target Side Duplications (TSD) and GenBank accession numbers are presented in Additional file 6.

### Sugarcane EST database screening

All full-length LTR-RTs were used as queries in a BLASTN search against EST sequences from the sugarcane cultivar SP80-3280. The ESTs were obtained using ENTREZ at NCBI http://www.ncbi.nlm.nih.gov/Entrez/. A total of 155,354 sugarcane ESTs were analyzed, all of them from the SUCEST (Sugarcane EST) project [44].

ESTs similar to LTR-RTs were assigned to a family according to the criteria based on Wicker et al. [5]: 80% coverage with 80% nucleotide identity.

The number of hits for each library was normalized by dividing the raw number of hits by the total number of valid reads. The normalized numbers of hits per library were then combined according to tissue type. The final number was multiplied by 100,000, so that in Figure 4 the X axis represents the number of ESTs per 100,000 transcripts from each tissue.

### Association of cDNAs to full-length LTR-RTs

Thirty manually curated sugarcane cDNAs related to LTR-RTs [20], described using an older nomenclature, were assigned to a family according to the same criteria used for the ESTs.

### RNA extraction and Reverse Transcriptase (RT) PCR Analysis

Leaf blade tissues were collected from one-month-old sugarcane plants (cultivar SP 80-3280) grown under greenhouse conditions. Mature eight-month-old plants of the same cultivar were used to obtain lateral buds. Stalk pieces with one bud (single eye sets) were planted in plastic trays containing a commercial planting mix (Plantmax, Eucatex, Brazil). After five days, developing buds were collected for RNA extraction. Two independent biological replicates were collected for leaf blade and lateral bud tissues. Total RNA was extracted using TRizol reagent (Invitrogen) according to the manufacturer's instructions.

Primers were designed within the reverse transcriptase domain using Primer3Plus [45] to amplify all known elements from a family. Total RNA was treated with DNAse I Amp Grade (Invitrogen) to remove any residual genomic DNA. Three micrograms of DNAse-treated RNA was used to generate the first strand cDNA using ImProm II Reverse Transcriptase (Promega) according to the manufacturer's instructions. The reaction mixture was placed in a GeneAmp9700 thermocycler (Applied Biosystems) and incubated at 16°C for 30 minutes, followed by 60 cycles of pulsed reverse transcription at 30°C for 30 seconds, 42°C for 30 seconds, and 50°C for one second. cDNA dilutions were used in PCR reactions as following: 1.0 μL of cDNA, 10 pmol of each primer, GoTaqmastermix, and 1 U of GoTaq DNA Polymerase (Promega) in a total volume of 25 μL. The reactions were placed in the thermocycler with the following conditions: 94°C for three minutes and appropriate cycle numbers of 94°C for 30 seconds, 55°C or 60°C for 30 seconds, and 72°C for 45 seconds. All reactions were repeated at least twice.

### Small RNA library construction and bioinformatic analysis

To evaluate the small RNA "landscape" of sugarcane LTR-RTs, we prepared a sRNA library from leaves of one-month old SP80-3280 sugarcane cultivar plants, grown under greenhouse conditions. Ten micrograms of total RNA, prepared using TRizol reagent (Invitrogen) according to the manufacturer's instructions, were used to generate sRNA library following Illumina's modified protocol. The sRNA fraction of 19-28 nt was purified by size fractionation on a 15% TBE-Urea polyacrylamide gel. A 5'-adenylated single-stranded adapter was first ligated to the 3'-end of the RNA using T4 RNA ligase without ATP, followed by a second single-stranded adapter ligation at the 5'-end of the RNA using T4 RNA ligase in the presence of ATP. The resulting products were fractioned on a 10% TBE-Urea polyacrylamide gel and then used for cDNA synthesis and PCR amplification. The resulting library was sequenced on an Illumina Genome Analyzer (GA-IIx) following the manufacturer's protocol available at http://www.fasteris.com.

A total of 4,388,665 20-25nt raw sequences were retrieved in a FASTQ formatted file and the adapter sequences were removed using Perl Scripts. After trimming of adapter sequences, the inserts were sorted into separate files according to their lengths. We used the program MAQ [46] to map 20-25 ntsRNA reads against sugarcane LTR-RT reference sequences (sequence 1 from each family). MAQ is a program that rapidly aligns short reads to reference genome sequences, and in this study we allowed 0-2 nt mismatches between the sRNA and LTR-RTs sequences. Three percent of the total library, that is, 131,641 high quality raw 20-25nt sequences matched against the sugarcane LTR-RT sequences. These sRNAs sequences have been submitted to the NCBI Gene Expression Omnibus database http://www.ncbi.nlm.nih.gov/geo under accession number GSE35143.

### Fluorescence in situ hybridization (FISH)

The distribution of the sugarcane LTR-RTs was analyzed by fluorescence *in situ *hybridization (FISH) on metaphase chromosomes. In order to compare the distribution of the LTR-RT relative to the centromere, a centromeric BAC [16] was also used as a probe. A single representative probe was used for each evolutionary lineage (Figure 3). The sequence of each probe was submitted as a query to a BLASTN analysis against a database of sugarcane cDNAs related to TEs identified in our lab [11,20] to check that, at 85% stringency, it would not hybridize against other elements.

All LTR-RT probes were 1.9 to 2.9 kb and covered the reverse transcriptase domain. For the *Ale1 *and *Ivana1 *families, probes were selected from previously reported cDNA sequences [11,20]. For *Ale1*, we used cDNA TE137 (GenBank accession [GenBank:JN786875]) and TE049 for *scIvana1 *(GenBank accession [GenBank:DQ115032]) on the basis of size (> 1.9 kb) and the presence of the reverse transcriptase domain. For all other lineages primers were designed from alignments of the RT domain using Primer3Plus [45]. All kits were used according to the manufacturer's instructions. The probe sequences were PCR amplified from R570 cultivar genomic DNA using Elongase (Invitrogen) or GoTaq (Promega) with 2 mM MgCl_2_, 0.2 mMdNTPs, 0.2 μM primers, 1 ng/μL genomic DNA and 0.025units/uL of Enzyme. Cycling conditions were as described in the Expand Long Template PCR System (Roche). The resulting amplicons were separated on 1% agarose, gel-purified using the NucleoSpin Extract II kit (Macherey Nagel), ligated into the pGEM T-Easy Vector (Promega), and cloned into DH10B electrocompetent cells according to standard procedures [47]. Minipreps from three clones from each PCR reaction and from the cDNA clones were prepared using standard alkaline precipitation methods [47], and sequenced using the vector primers M13F/R. In order to obtain a probe that consisted of just the probe, one miniprep for each lineage was diluted 1:1000 and used as template in 100 μL PCR reaction with M13F/R primers to amplify the insert only, using GoTaq (Promega) in same reaction conditions as above, but with the following cycling conditions, initial denaturation 95°C 3 min, 35cycles of 95°C 1 min, 55°C 30 sec, 72°C 2 min, followed by a final extension of 72°C for 3 min. The resulting amplicons were separated on 1% agarose, gel purified using the NucleoSpin Extract II kit (Macherey Nagel) and quantified using a NanoDrop Spectrometer (ThermoScientific). For the centromeric BAC probe, BAC DNA was extracted using the Large-Construct Kit (Qiagen).

Between 350-700 ng of probe DNA was used in a 20 μL nick translation reaction with Digoxigenin (DIG)-11-dUTP (Invitrogen) or Biotin-16-dUTP (Invitrogen) and the NT mix (Roche). Labeling efficiency was tested according to Heslop (2000) [48] (protocol 4.7). The probe was only used if the 1:1000 dilution was clearly visible.

Sections of sugarcane stalk from the cultivar SP80-3280 were planted in a mixture of 1/2 soil 1/2 vermiculite, root tips harvested within 1-3 days and placed directly into 2 mM 8-hydroxyquinoline for 6 hours at 18°C. Next, they were transferred to 3:1 ethanol:acetic acid fixative and stored at -20°C. Root samples were prepared according to Heslop(2000) [48], protocol 5.3, except that they were digested in either 2% cellulase/0.2% macerozyme/20% pectinase or 1% cellulase/0.2% macerozyme for 2 1/2 to 3 hours (depending on how large the root tip was) at 37°C.

Hybridization and detection was performed according to Heslop (2000) [48] using protocols 8.1, 8.4, 9.1 and 9.2, with the following conditions: the slide was dried for 30 min at 50-60°C and pretreated with both RNAse A and pepsin (20 min at 37°C); 1 μL of each labeled probe was added to a 20 μL hybridization mix of 50% formamide/2xSSC/10% dextran sulphate/1%SDS; the slide was denatured in 50 mL of 70% formamide/2xSSC at 70°C for 2 min and then dehydrated through an ice-cold ethanol series (70%, 85%, 100% ethanol); washes were 80-82% stringent, 20% formamide with 0.1 or 0.2 xSSC at 42°C; DIG-labeled probes were detected with anti-digoxigenin-rhodamine (Roche), biotin-labeled with NeutrAvidin-Oregon Green-488 (Molecular Probes).

The slide was stained with DAPI, observed and photographed with an Zeiss AxioPlan2 microscope and captured using an Axiocam MR camera and the Isis Fluorescence Imaging System (MetaSystems). Nine to 25 metaphases were photographed for each probe. Slides were stripped by carefully removing the immersion oil, soaked in 4xSSC/0.1% Tween 20 at 37°C until the coverslip floated off, transferred to fresh 4xSSC/0.1% Tween 20 for 3 hours with gentle shaking, transferred to 3:1 ethanol:acetic acid fixative for 30 min and then dehydrated through an ethanol series (70%, 85%, 100% ethanol) for 5 min each at room temperature and air dried for 1 hour.

## Competing interests

The authors declare that they have no competing interests

## Authors' contributions

The project was designed by MAVS, DSD and GMQC, and co-ordinated by MAVS. DSD, GMQC and CJM did the bio-informatic analyses. CJM carried out the FISH assays. FTSN and RV made the sRNA library and did the sRNA analyses. CSA did the RT-PCRs. DSD, GMQC, CJM, FTSN, RV and MAVS wrote the manuscript. All authors read and approved the final manuscript.

## Supplementary Material

Additional file 1 ***Gypsy *and *Copia *Maximum Likelihood phylogenies**. Maximum-likelihood phylogeny of *Gypsy *families (Figure 1) based on reverse transcriptase. Sequences from the Gypsy database [8] are denoted with a plus symbol, those from Du et al. [7] with a star. Maximum-likelihood phylogeny of *Copia *families (Figure 2) based on reverse transcriptase. Sequences from the Gypsy database [8] are denoted with a plus symbol, those from Wicker and Keller [6] with a star. Robustness of the nodes was estimated by 500 bootstrap replications. Bootstrap values below 50 are not shown. Click here for file

Additional file 2 **Fluorescence in situ hybridization with the DEL probe**. Figure of fluorescence *in situ *hybridization using the *Del *probe, prepared and labelled twice, once with Digoxigenin (DIG) and detected with Anti-DIG-Rhodomine (red signal) and once with Biotin and detected with NeutrAvidin-Oregon Green(green signal). The probes were hybridized to the same slide in consecutive FISH experiments under the same conditions. The same pattern was observed for both probes, suggesting that the signal was real, and the same FISH conditions was used for all LTR-RT probe. Click here for file

Additional file 3 **Assignment of previously described cDNA sequences to LTR-RT families**. Thirty manually curated sugarcane cDNAs related to LTR-RTs [20] were assigned to a family according Wicker et al. [5]: 80% coverage with 80% nucleotide identity. Click here for file

Additional file 4 **sRNA mapping to individual LTR-RT elements**. Mapping of sRNAs within each LTR-RT family (Figures 1 to 7). 20-22nt sRNAs are represented as blue lines, 23-25ntsRNAs as red lines. Each figure shows a different lineage, and includes all the families of that lineage. A scaled schematic (also shown in Figures 1 and 2), is shown below each sRNA map. Click here for file

Additional file 5 **Total 20-22nt and 23-25nt sRNA counts for each LTR-RT family**. Total 20-22nt (black) and 23-25nt (grey) sRNA counts for each LTR-RT family, with a mismatch of 2nt allowed. Click here for file

Additional file 6 **LTR-RT information**. Name assigned in this paper, pre-existing name from [14,20], GenBank accession number, size of full-length element, length of 5' and 3' LTRs, and sequence of 5 and 3' TSDs, for individual LTR-RT sequences. Click here for file
