# Supplementary material for: Analysis of plant LTR-retrotransposons at the fine-scale family level reveals individual molecular patterns
Source: BMC Genomics. 2012 Apr 16;13:137. doi: 10.1186/1471-2164-13-137 (PMC3352295; doi:10.1186/1471-2164-13-137)
Supplement: Additional file 1 — Gypsy and Copia Maximum Likelihood phylogenies. Maximum-likelihood phylogeny of Gypsy families (Figure 1) based on reverse transcriptase. Sequences from the Gypsy database [8] are denoted with a plus symbol, those from Du et al. [7] with a star. Maximum-likelihood phylogeny of Copia families (Figure 2) based on reverse transcriptase. Sequences from the Gypsy database [8] are denoted with a plus symbol, those from Wicker and Keller [6] with a star. Robustness of the nodes was estimated by 500 bootstrap replications. Bootstrap values below 50 are not shown. [file 1471-2164-13-137-S1.PPTX]

## Slide 1
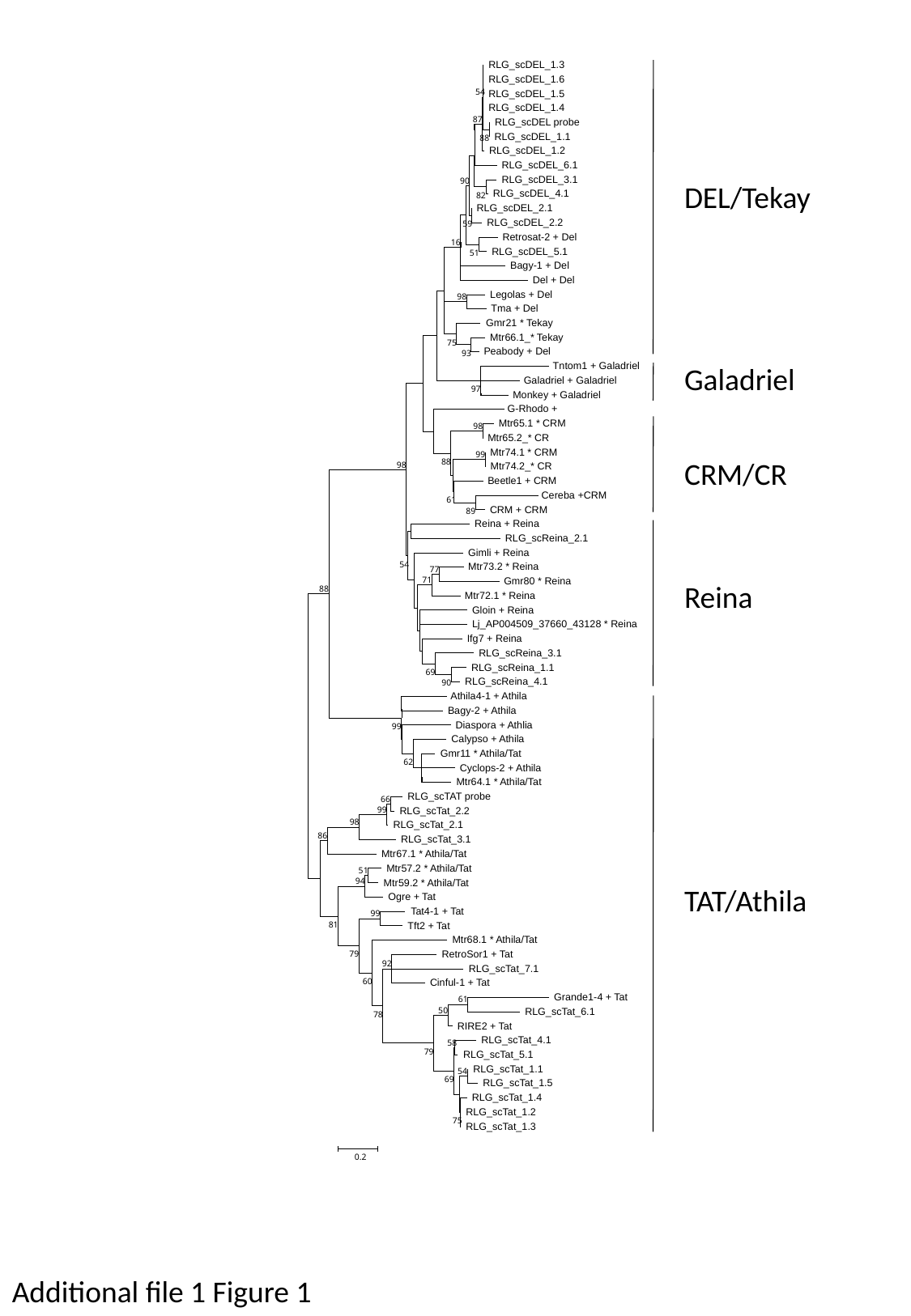

RLG_scDEL_1.3
 RLG_scDEL_1.6
 RLG_scDEL_1.5
 RLG_scDEL_1.4
 RLG_scDEL probe
 RLG_scDEL_1.1
 RLG_scDEL_1.2
 RLG_scDEL_6.1
 RLG_scDEL_3.1
 RLG_scDEL_4.1
 RLG_scDEL_2.1
 RLG_scDEL_2.2
 Retrosat-2 + Del
 RLG_scDEL_5.1
 Bagy-1 + Del
 Del + Del
 Legolas + Del
 Tma + Del
 Gmr21 * Tekay
 Mtr66.1_* Tekay
 Peabody + Del
 Tntom1 + Galadriel
 Galadriel + Galadriel
 Monkey + Galadriel
 G-Rhodo +
 Mtr65.1 * CRM
 Mtr65.2_* CR
 Mtr74.1 * CRM
 Mtr74.2_* CR
 Beetle1 + CRM
 Cereba +CRM
 CRM + CRM
 Reina + Reina
 RLG_scReina_2.1
 Gimli + Reina
 Mtr73.2 * Reina
 Gmr80 * Reina
 Mtr72.1 * Reina
 Gloin + Reina
 Lj_AP004509_37660_43128 * Reina
 Ifg7 + Reina
 RLG_scReina_3.1
 RLG_scReina_1.1
 RLG_scReina_4.1
 Athila4-1 + Athila
 Bagy-2 + Athila
 Diaspora + Athlia
 Calypso + Athila
 Gmr11 * Athila/Tat
 Cyclops-2 + Athila
 Mtr64.1 * Athila/Tat
 RLG_scTAT probe
 RLG_scTat_2.2
 RLG_scTat_2.1
 RLG_scTat_3.1
 Mtr67.1 * Athila/Tat
 Mtr57.2 * Athila/Tat
 Mtr59.2 * Athila/Tat
 Ogre + Tat
 Tat4-1 + Tat
 Tft2 + Tat
 Mtr68.1 * Athila/Tat
 RetroSor1 + Tat
 RLG_scTat_7.1
 Cinful-1 + Tat
 Grande1-4 + Tat
 RLG_scTat_6.1
 RIRE2 + Tat
 RLG_scTat_4.1
 RLG_scTat_5.1
54
87
88
DEL/Tekay
90
82
59
16
51
98
75
93
Galadriel
97
98
CRM/CR
99
88
98
61
89
54
77
Reina
71
88
69
90
99
62
66
99
98
86
51
TAT/Athila
94
99
81
79
92
60
61
50
78
58
79
 RLG_scTat_1.1
54
69
 RLG_scTat_1.5
 RLG_scTat_1.4
 RLG_scTat_1.2
75
 RLG_scTat_1.3
0.2
Additional file 1 Figure 1

## Slide 2
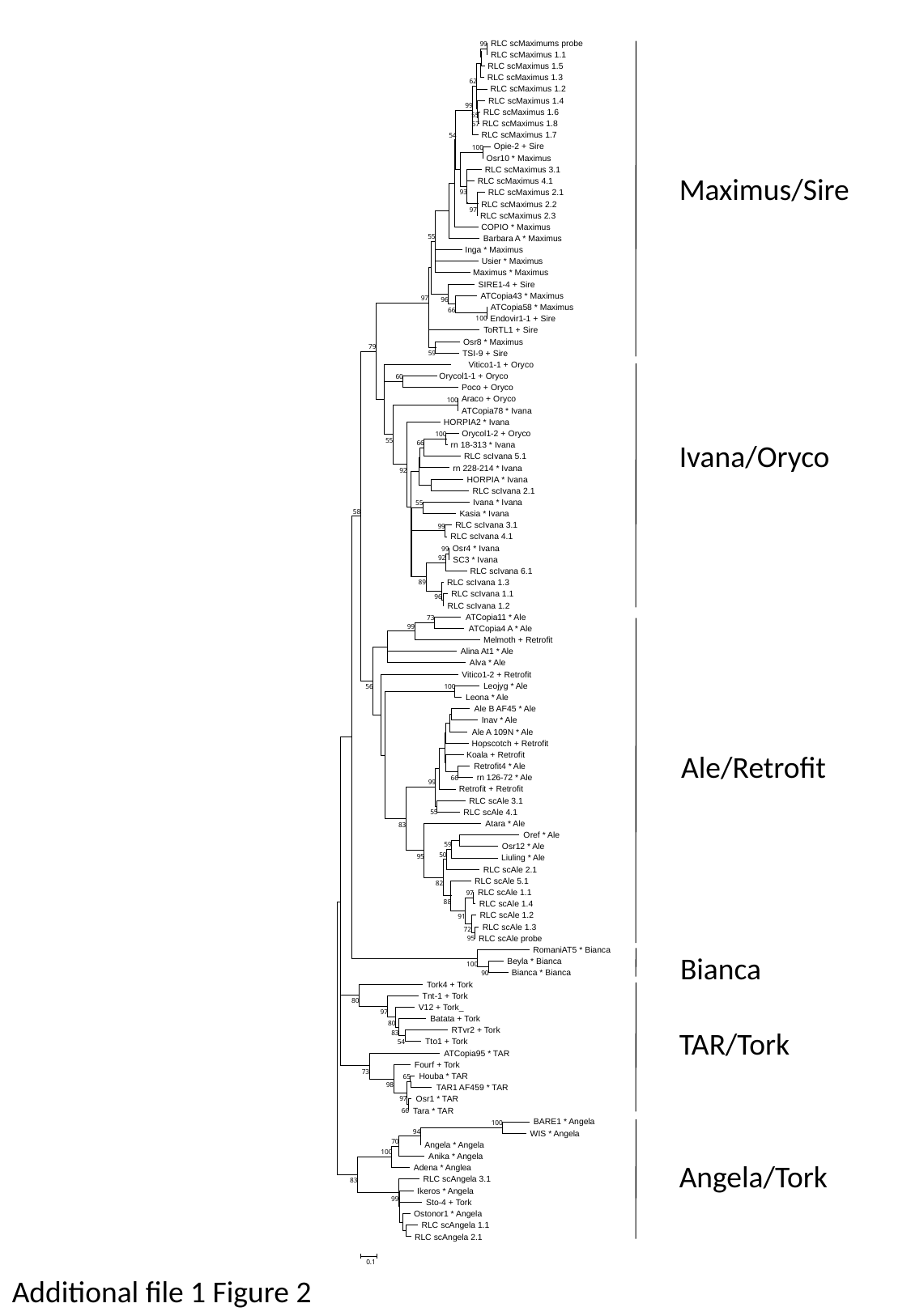

RLC scMaximums probe
 RLC scMaximus 1.1
 RLC scMaximus 1.5
 RLC scMaximus 1.3
 RLC scMaximus 1.2
 RLC scMaximus 1.4
 RLC scMaximus 1.6
 RLC scMaximus 1.8
 RLC scMaximus 1.7
 Opie-2 + Sire
 Osr10 * Maximus
 RLC scMaximus 3.1
 RLC scMaximus 4.1
 RLC scMaximus 2.1
 RLC scMaximus 2.2
 RLC scMaximus 2.3
 COPIO * Maximus
 Barbara A * Maximus
 Inga * Maximus
 Usier * Maximus
 Maximus * Maximus
 SIRE1-4 + Sire
 ATCopia43 * Maximus
 ATCopia58 * Maximus
 Endovir1-1 + Sire
 ToRTL1 + Sire
 Osr8 * Maximus
 TSI-9 + Sire
 Vitico1-1 + Oryco
 Orycol1-1 + Oryco
 Poco + Oryco
 Araco + Oryco
 ATCopia78 * Ivana
 HORPIA2 * Ivana
 Orycol1-2 + Oryco
 rn 18-313 * Ivana
 RLC scIvana 5.1
 rn 228-214 * Ivana
 HORPIA * Ivana
 RLC scIvana 2.1
 Ivana * Ivana
 Kasia * Ivana
 RLC scIvana 3.1
 RLC scIvana 4.1
 Osr4 * Ivana
 SC3 * Ivana
 RLC scIvana 6.1
 RLC scIvana 1.3
 RLC scIvana 1.1
 RLC scIvana 1.2
 ATCopia11 * Ale
 ATCopia4 A * Ale
 Melmoth + Retrofit
 Alina At1 * Ale
 Alva * Ale
 Vitico1-2 + Retrofit
 Leojyg * Ale
 Leona * Ale
 Ale B AF45 * Ale
 Inav * Ale
 Ale A 109N * Ale
 Hopscotch + Retrofit
 Koala + Retrofit
 Retrofit4 * Ale
 rn 126-72 * Ale
 Retrofit + Retrofit
 RLC scAle 3.1
 RLC scAle 4.1
 Atara * Ale
99
62
99
55
67
54
100
93
97
55
97
96
66
100
79
59
60
100
100
55
66
92
55
58
99
99
92
89
96
73
99
56
100
66
99
55
83
 Oref * Ale
59
 Osr12 * Ale
50
95
 Liuling * Ale
 RLC scAle 2.1
 RLC scAle 5.1
82
 RLC scAle 1.1
97
88
 RLC scAle 1.4
 RLC scAle 1.2
91
 RLC scAle 1.3
72
 RLC scAle probe
95
 RomaniAT5 * Bianca
 Beyla * Bianca
100
 Bianca * Bianca
90
 Tork4 + Tork
 Tnt-1 + Tork
80
 V12 + Tork_
97
 Batata + Tork
80
 RTvr2 + Tork
83
 Tto1 + Tork
54
 ATCopia95 * TAR
 Fourf + Tork
73
 Houba * TAR
65
98
 TAR1 AF459 * TAR
 Osr1 * TAR
97
 Tara * TAR
66
 BARE1 * Angela
 WIS * Angela
 Angela * Angela
100
 Anika * Angela
 Adena * Anglea
 RLC scAngela 3.1
83
 Ikeros * Angela
99
 Sto-4 + Tork
 Ostonor1 * Angela
 RLC scAngela 1.1
 RLC scAngela 2.1
100
94
70
0.1
Maximus/Sire
Ivana/Oryco
Ale/Retrofit
Bianca
TAR/Tork
Angela/Tork
Additional file 1 Figure 2
