# Supplementary figures and images for: Analysis of plant LTR-retrotransposons at the fine-scale family level reveals individual molecular patterns
Source: BMC Genomics. 2012 Apr 16;13:137. doi: 10.1186/1471-2164-13-137 (PMC3352295; doi:10.1186/1471-2164-13-137)

## Slide 1
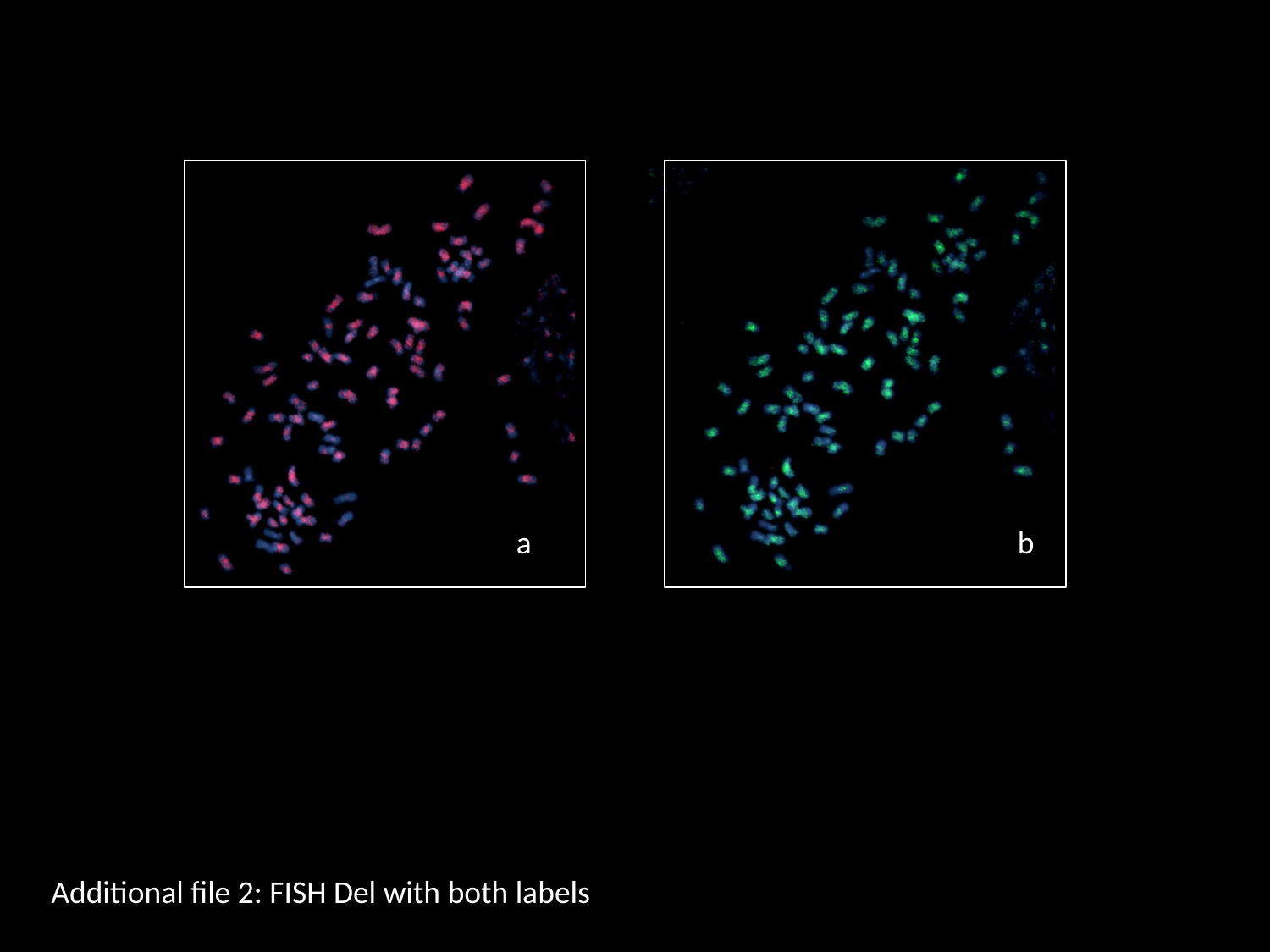

a
b
Additional file 2: FISH Del with both labels

Supplement: Additional file 2 — Fluorescence in situ hybridization with the DEL probe. Figure of fluorescence in situ hybridization using the Del probe, prepared and labelled twice, once with Digoxigenin (DIG) and detected with Anti-DIG-Rhodomine (red signal) and once with Biotin and detected with NeutrAvidin-Oregon Green(green signal). The probes were hybridized to the same slide in consecutive FISH experiments under the same conditions. The same pattern was observed for both probes, suggesting that the signal was real, and the same FISH conditions was used for all LTR-RT probe. [file 1471-2164-13-137-S2.PPTX]

## Slide 1
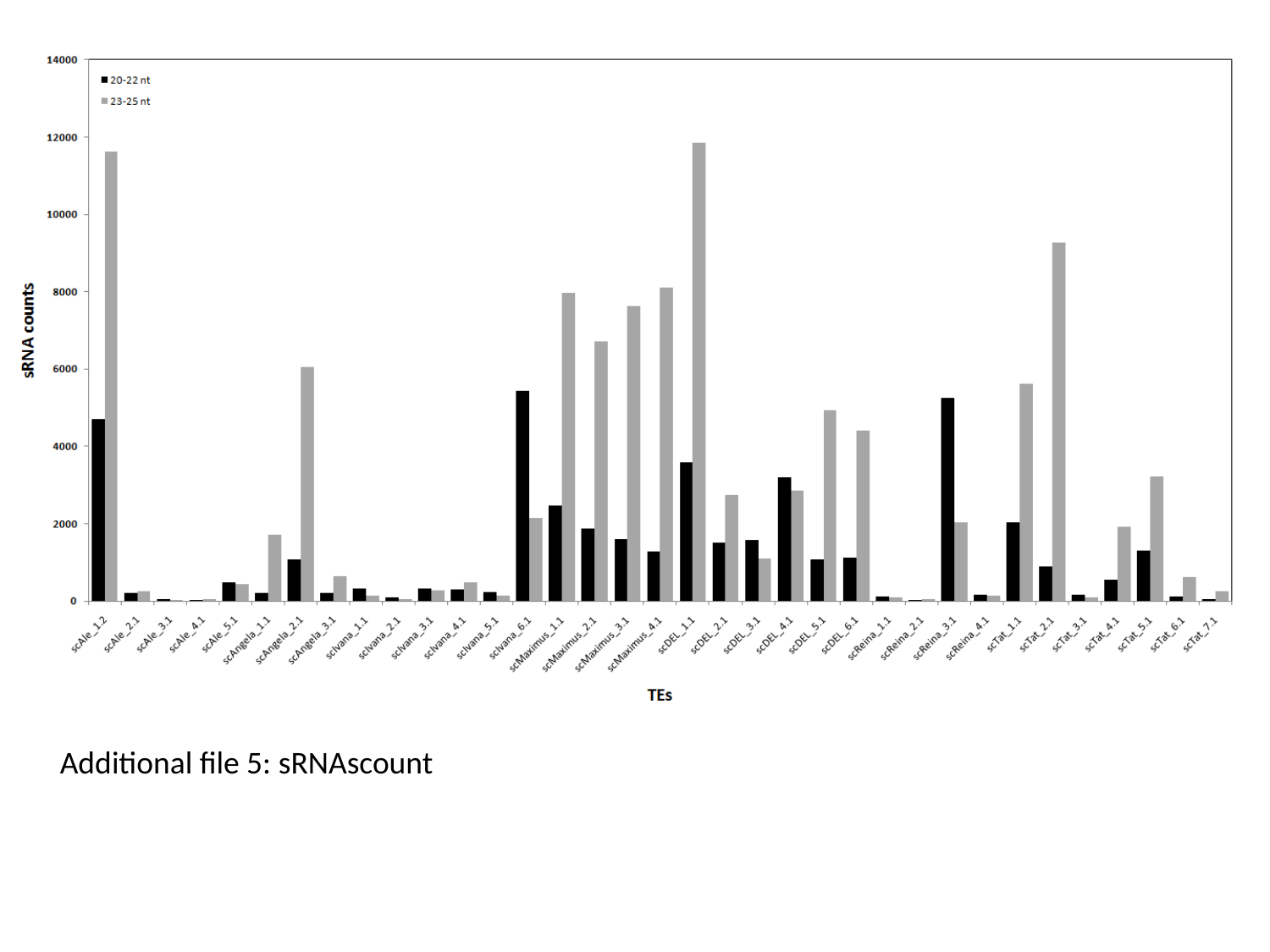

Additional file 5: sRNAscount

Supplement: Additional file 5 — Total 20-22nt and 23-25nt sRNA counts for each LTR-RT family. Total 20-22nt (black) and 23-25nt (grey) sRNA counts for each LTR-RT family, with a mismatch of 2nt allowed. [file 1471-2164-13-137-S5.PPTX]
