# Supplementary material for: Analysis of plant LTR-retrotransposons at the fine-scale family level reveals individual molecular patterns
Source: BMC Genomics. 2012 Apr 16;13:137. doi: 10.1186/1471-2164-13-137 (PMC3352295; doi:10.1186/1471-2164-13-137)
Supplement: Additional file 4 — sRNA mapping to individual LTR-RT elements. Mapping of sRNAs within each LTR-RT family (Figures 1 to 7). 20-22nt sRNAs are represented as blue lines, 23-25ntsRNAs as red lines. Each figure shows a different lineage, and includes all the families of that lineage. A scaled schematic (also shown in Figures 1 and 2), is shown below each sRNA map. [file 1471-2164-13-137-S4.PPTX]

## Slide 1
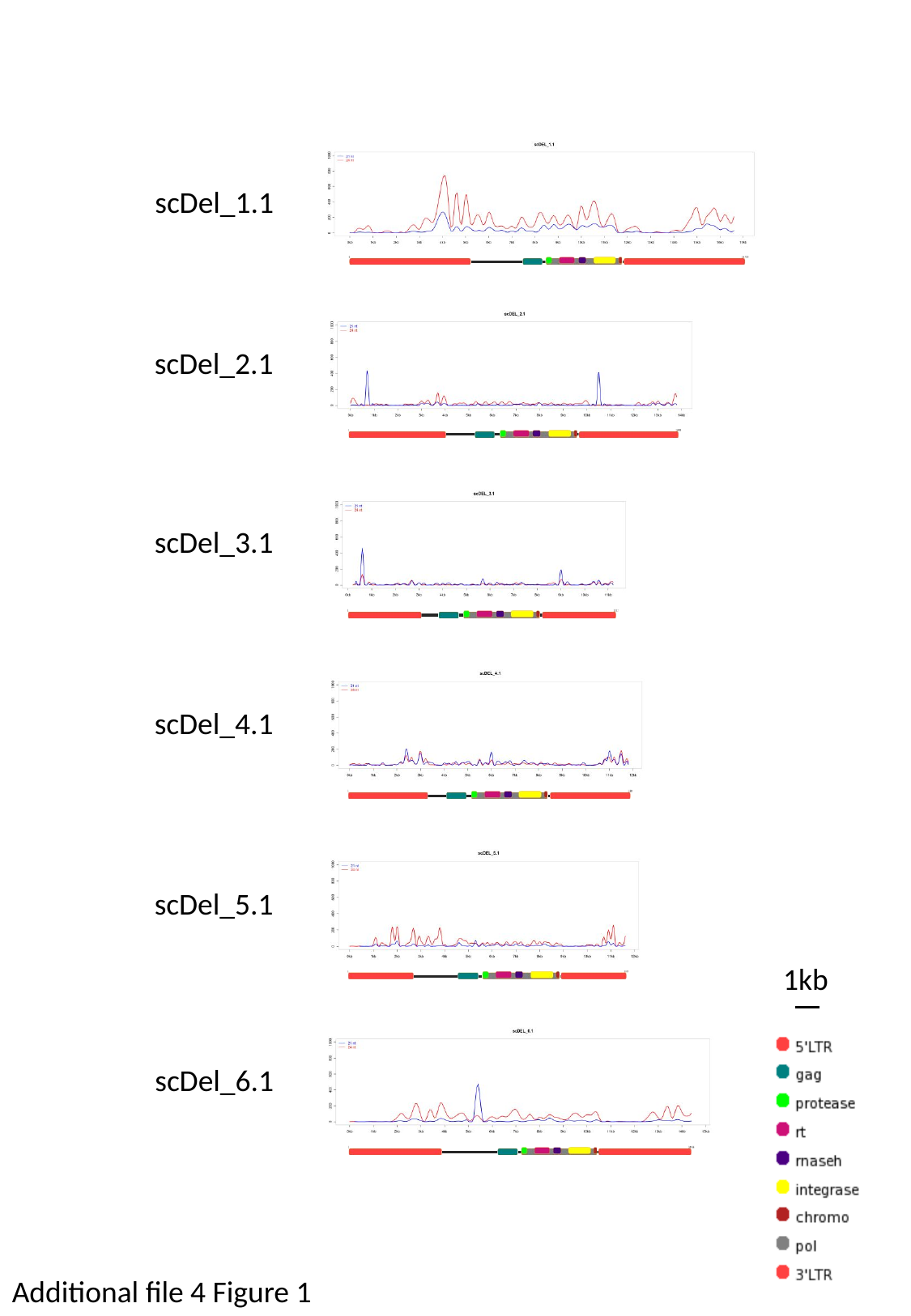

scDel_1.1
scDel_2.1
scDel_3.1
scDel_4.1
scDel_5.1
scDel_6.1
1kb
Additional file 4 Figure 1

## Slide 2
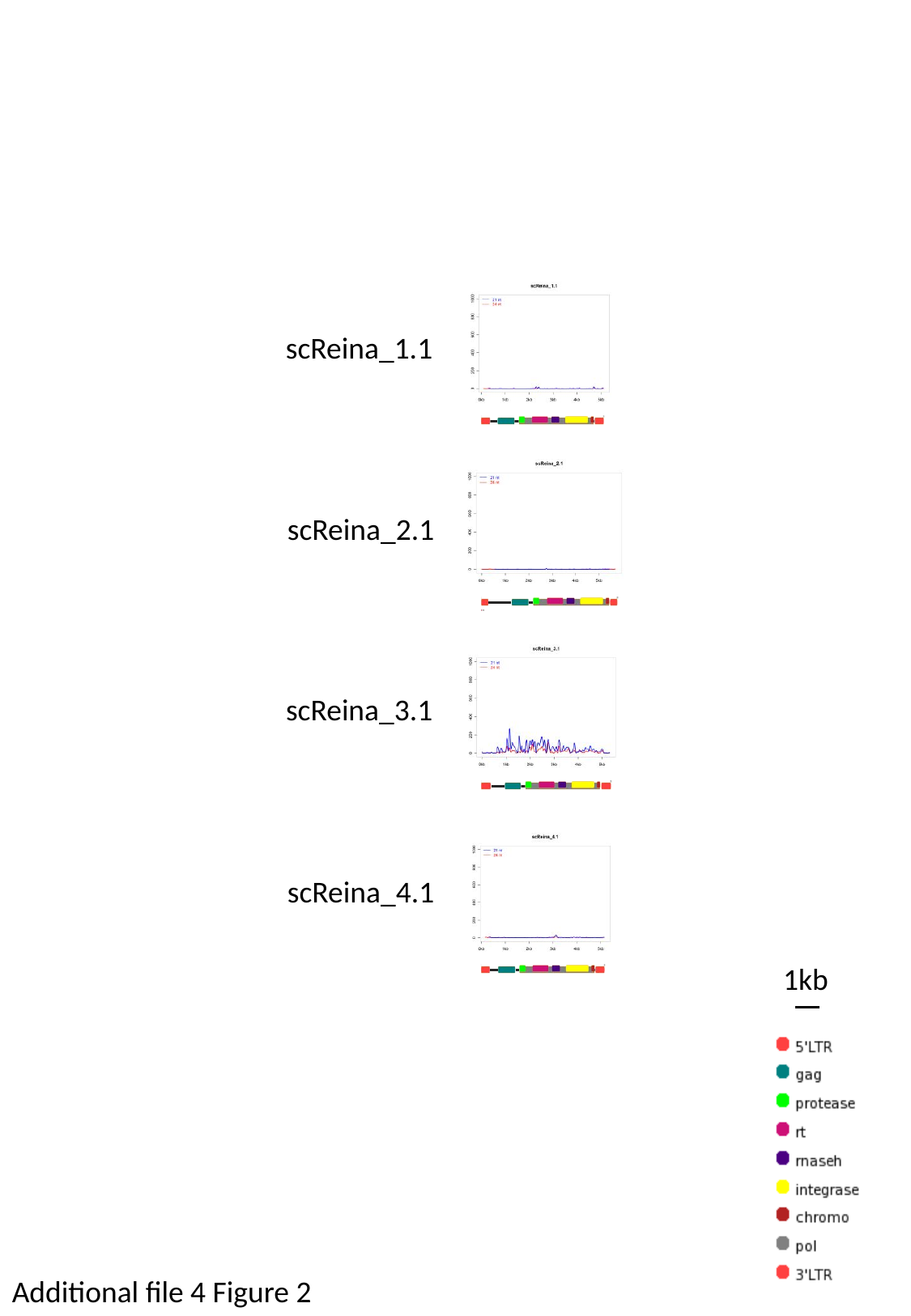

scReina_1.1
scReina_2.1
scReina_3.1
scReina_4.1
1kb
Additional file 4 Figure 2

## Slide 3
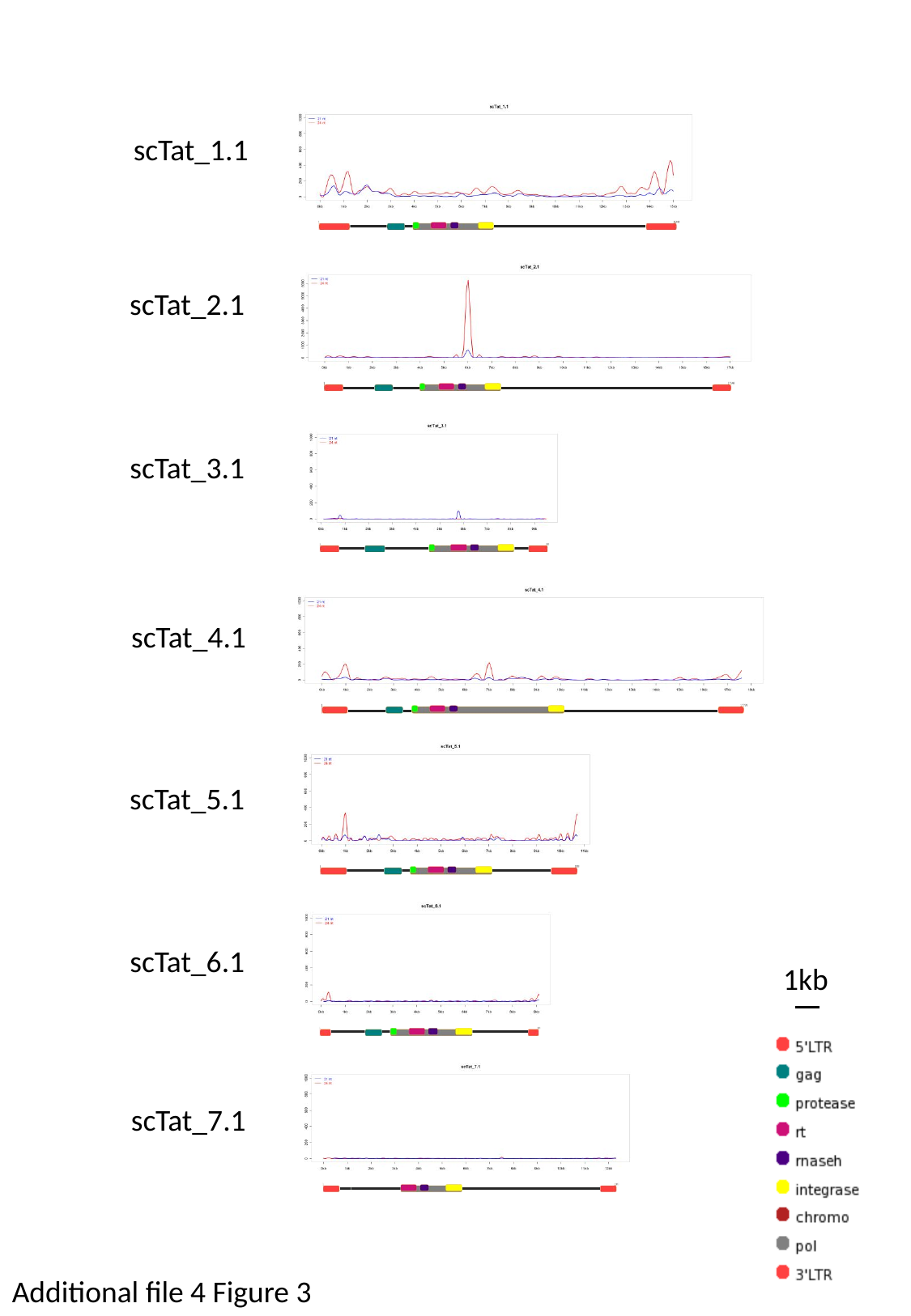

scTat_1.1
scTat_2.1
scTat_3.1
scTat_4.1
scTat_5.1
scTat_6.1
scTat_7.1
1kb
Additional file 4 Figure 3

## Slide 4
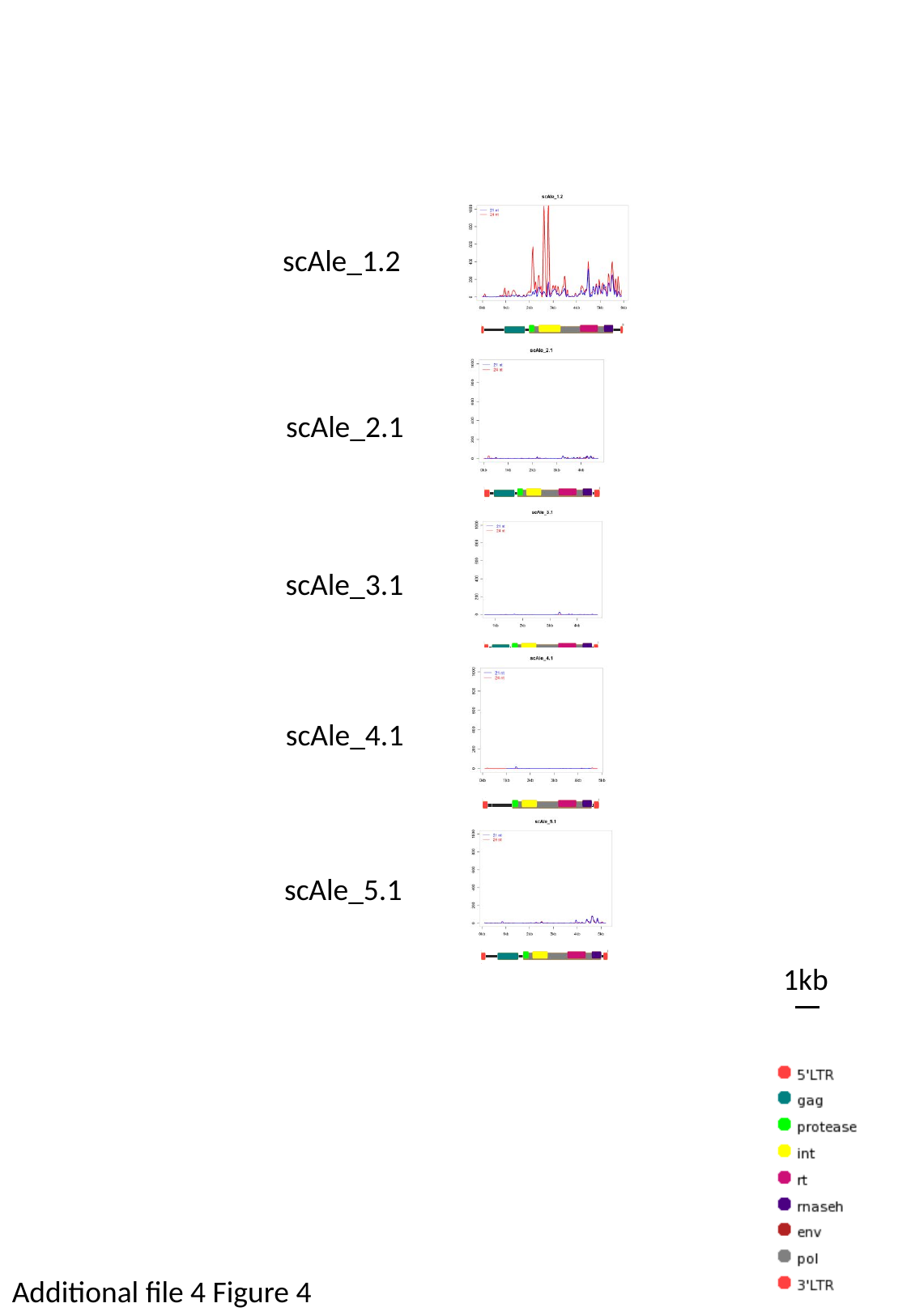

scAle_1.2
scAle_2.1
scAle_3.1
scAle_4.1
scAle_5.1
1kb
Additional file 4 Figure 4

## Slide 5
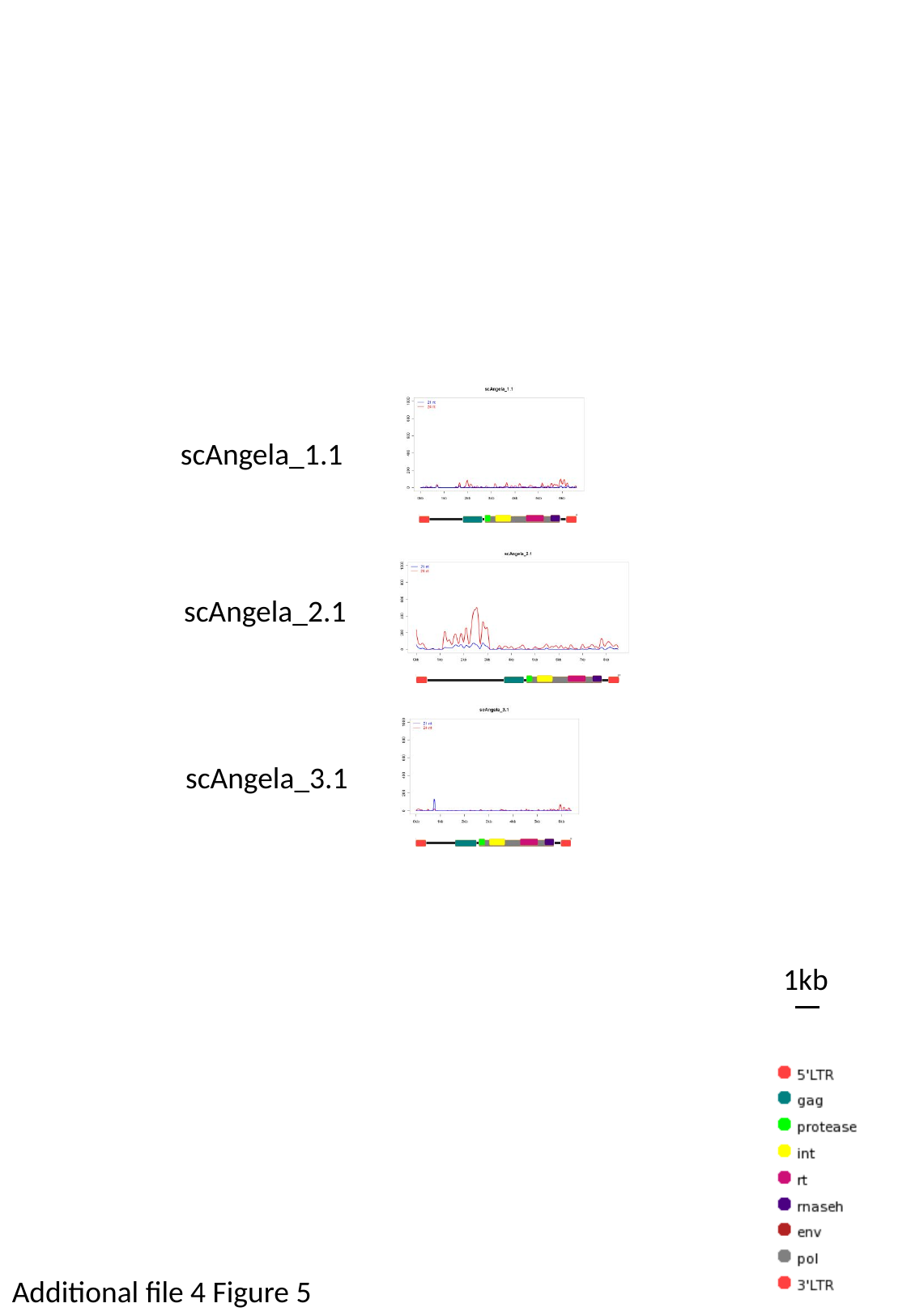

scAngela_1.1
scAngela_2.1
scAngela_3.1
1kb
Additional file 4 Figure 5

## Slide 6
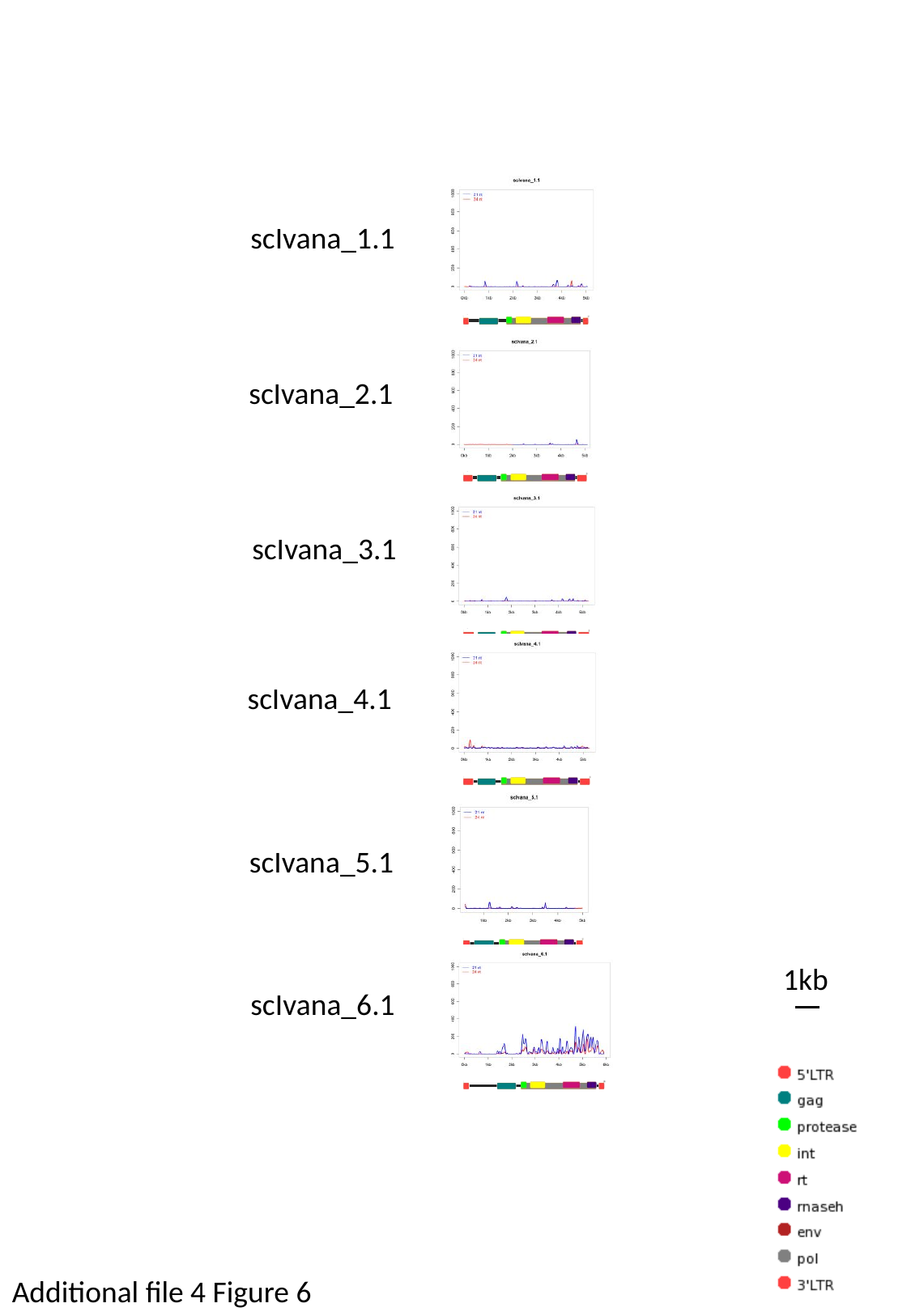

scIvana_1.1
scIvana_2.1
scIvana_3.1
scIvana_4.1
scIvana_5.1
scIvana_6.1
1kb
Additional file 4 Figure 6

## Slide 7
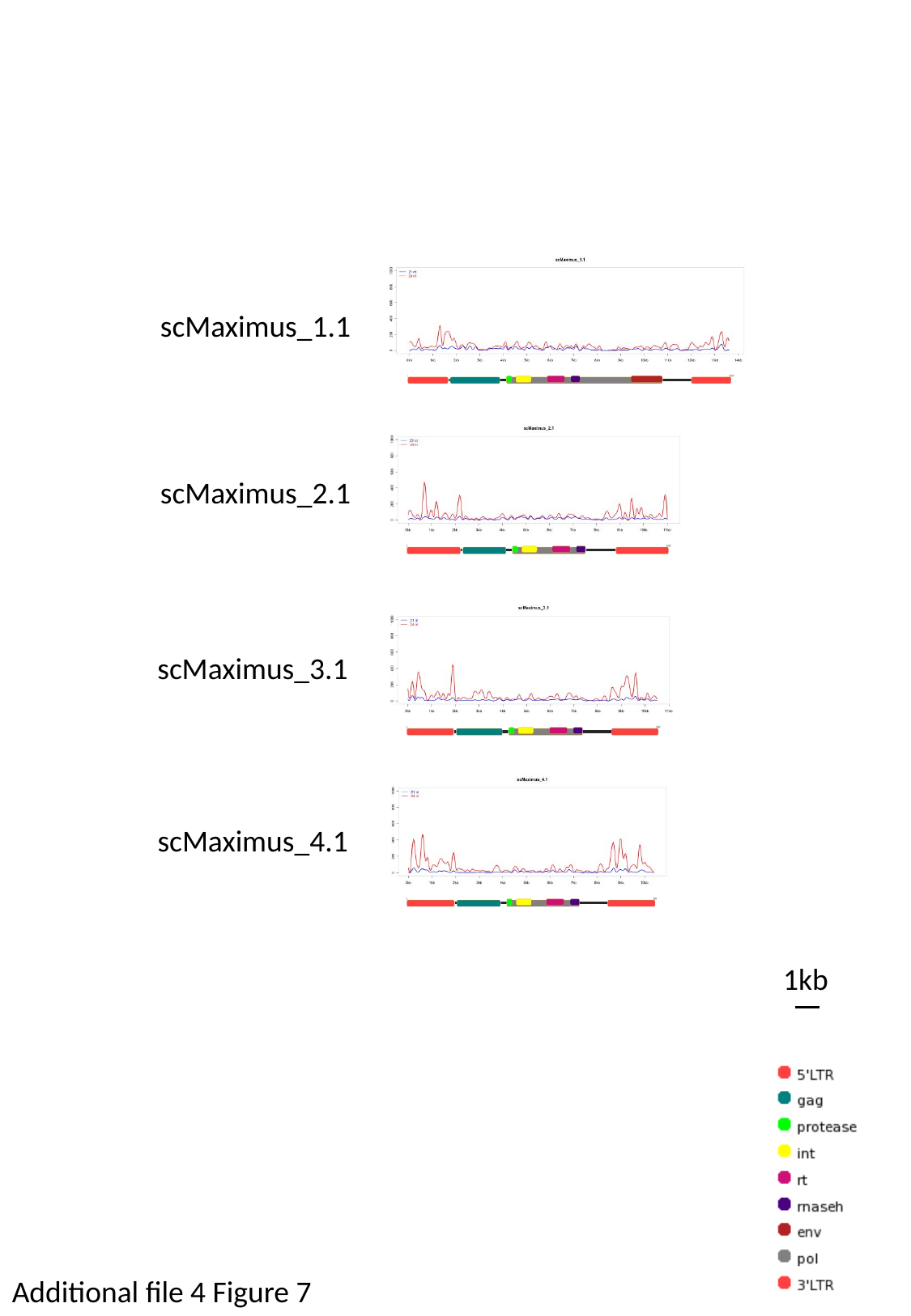

scMaximus_1.1
scMaximus_2.1
scMaximus_3.1
scMaximus_4.1
1kb
Additional file 4 Figure 7
